# Predictive modeling of biodegradation pathways using transformer architectures

**DOI:** 10.1186/s13321-025-00969-7

**Published:** 2025-02-17

**Authors:** Liam Brydon, Kunyang Zhang, Gillian Dobbie, Katerina Taškova, Jörg Simon Wicker

**Affiliations:** 1 School of Computer Science, University of Auckland, Auckland, New Zealand; 2 Eawag-Swiss Federal Institute of Aquatic Science and Technology, Dübendorf, Switzerland; 3 University of Zürich, Zürich, Switzerland; 4 enviPath UG & Co. KG, Ockenheim, Germany

**Keywords:** Cheminformatics, Biodegradation, Transformer, Transfer-learning, Product prediction

## Abstract

In recent years, the integration of machine learning techniques into chemical reaction product prediction has opened new avenues for understanding and predicting the behaviour of chemical substances. The necessity for such predictive methods stems from the growing regulatory and social awareness of the environmental consequences associated with the persistence and accumulation of chemical residues. Traditional biodegradation prediction methods rely on expert knowledge to perform predictions. However, creating this expert knowledge is becoming increasingly prohibitive due to the complexity and diversity of newer datasets, leaving existing methods unable to perform predictions on these datasets. We formulate the product prediction problem as a sequence-to-sequence generation task and take inspiration from natural language processing and other reaction prediction tasks. In doing so, we reduce the need for the expensive manual creation of expert-based rules.

## Scientific contribution

We contribute the first study of the transformer’s ability to predict biodegradation reactions. Our proposed method can more accurately and efficiently predict biodegradation reactions on more compounds than existing methods. We also contribute a framework for evaluating transformer product prediction methods that can better illustrate the method’s performance and is more suitable for comparison to other methods.

## Introduction

Awareness of the environmental impact of commonly used chemicals is growing globally, with many regulatory bodies pushing for the development of new environmentally friendly chemicals [1]. These regulations can require those developing any chemical that could be released into the environment to model how these chemicals biodegrade to ensure that they create no harmful byproducts. Therefore, modeling the biodegradation of chemicals is essential for designing new chemicals that do not harm the environment and for compliance with new regulatory standards [2]. Traditionally, this has meant arduously setting up and repeating experiments in a chemistry laboratory, which is time-consuming and expensive.

**Fig. 1:**
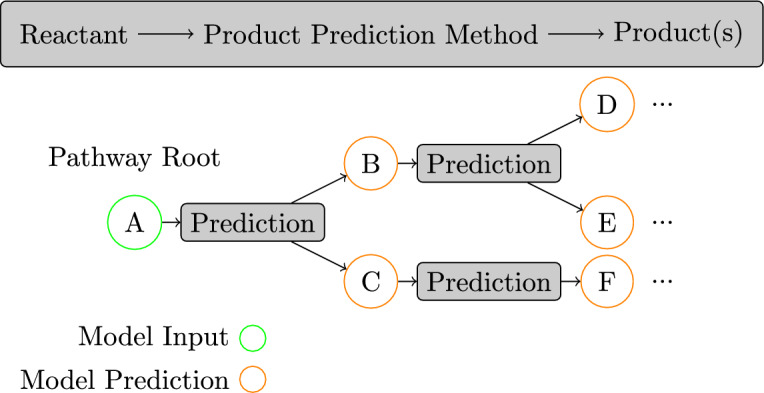
How pathway prediction is performed. A root molecule is given to a prediction method which recursively predicts products from reactants until an arbitrary stopping condition is reached

In recent years, the development of efficient and accurate biodegradation prediction methods has dramatically improved the speed of novel chemical development [3, 4]. Doing so allows those developing novel chemicals to identify potentially harmful compounds before committing significant resources to running laboratory experiments on a candidate chemical. The biodegradation process can be represented as a directed graph, with the root compound being the initial compound that degrades into one or more compounds, which in turn further degrades. Each edge in this graph represents a reaction, with the parent nodes being the *reactants* and the children being the *products*. This graph is known as the biodegradation *pathway*. Figure 1 illustrates the pathway prediction process starting from a supplied root compound.

Previous efforts to streamline the laboratory experiments focused on crafting expert rules for transformations to describe common reaction types in the degradation pathway [3]. These rules outline common patterns and substructures of compounds and detail how those structures change during a reaction. However, these rules are susceptible to being overly general, resulting in numerous rules applying to a single compound. This results in a combinatorial explosion of predicted products, as every rule will predict one or more products [5]. The combinatorial explosion becomes especially problematic when analysing entire biotransformation pathways, as the number of products grows exponentially at each level in the graph. We previously solved the combinatorial explosion by training a machine learning model to predict the likelihoods of the products, thus allowing unlikely products to be pruned [4]. However, this method still requires the expert-rules to be present for a given dataset.

Most recently, methods have been proposed for generating expert-style rules from the biodegradation data [6]. This is motivated by the growth in biodegradation datasets, making it challenging to create rules manually. Generated rules can be substituted for or added to the expert rules used in our previous method [4], with the subsequent models showing performance improvements [6]. However, utilising transformation rules comes with limitations. Notably, generated or expert-made existing rules have low *coverage*, meaning they cannot be applied to all biodegradation reactions, thus making prediction impossible on some compounds. This is due to the rule syntax only being able to capture limited complexity from the reaction diversity. Additionally, using these rules to simulate a reaction is computationally intensive, making existing prediction resources slow to use [7]. To address these limitations we introduce enviFormer, a purely data-driven method for predicting biodegradation products and pathways that does not require rules. Using enviFormer allows for more efficient and accurate predictions to be performed on biodegradation datasets compared to existing methods.

For the development and evaluation of enviFormer, we use enviPath, the largest source of biodegradation reaction data [8, 9]. It contains approximately 4000 biodegradation reactions across three datasets: Biocatalysis/Biodegradation Database (BBD), Soil and Sludge.[10–12].

**Fig. 2:**
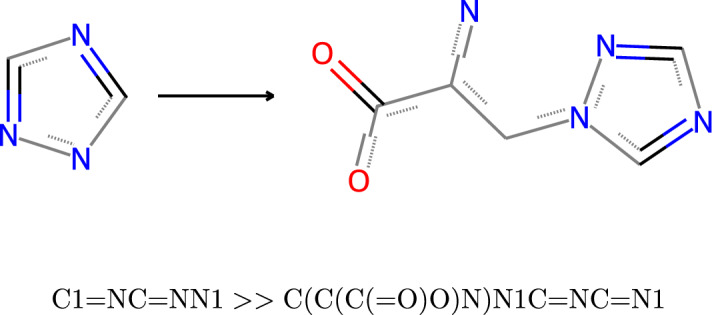
An example reaction SMILES where 1,2,4-triazole degrades into Triazole-alanine

We draw inspiration from existing research that has used transformer-based methods [13] on generic chemical reaction datasets [14, 15]. Additionally, we introduce transfer-learning [16] to biodegradation datasets, where we first train our method on a large dataset of general chemical reactions before refining it on the smaller biodegradation datasets. The application of transfer-learning to small reaction datasets has shown promising results on single type reaction datasets such as the Baeyer-Villiger and Heck datasets [17, 18]. Like the existing transformer-based methods, we formulate the product prediction task as a sequence-to-sequence translation task drawing inspiration from the field of Natural Language Processing (NLP). To represent compounds, we use the Simplified Molecular Input Line Entry System (SMILES) [19], a sequence representation of the 3D molecular structure. Reactions are represented as reaction SMILES, made of two SMILES separated by two greater than (i.e. $\gg$) characters. Figure 2 shows an example reaction, including its visual representation and the reaction SMILES. EnviFormer aims to generate a SMILES sequence defining the product(s) the input SMILES sequence will produce during biodegradation. Thus, the core of our evaluation will be checking that the standardised predicted SMILES are equivalent to the standardised SMILES of the products stored in the dataset.

For evaluating our method, we introduce a novel transformer evaluation metric by proposing a framework for assessing transformer-based methods that allows for a fair comparison with existing biodegradation prediction methods. Current transformer-based methods use a naïve Top-K accuracy evaluation methodology that does not examine the actual likelihood the model gives to a prediction and ignores the drop in precision from predicting multiple product sets [14, 15, 17]. Instead, we look at applying a threshold to accept or reject model predictions, allowing us to produce Precision–Recall curves that better illustrate our method’s capabilities.

Overall, this paper evaluates the ability of transformer models to learn and predict products of biodegradation reactions and pathways. To do so, we propose enviFormer, which can accurately and efficiently predict biodegradation reaction products. Unlike rule-based approaches, our approach can perform product prediction on any input molecule, improving the coverage of biodegradation prediction methods. We also contribute a framework for evaluating any product prediction method beyond biodegradation. The framework gives more insight into the method’s performance than the common Top-K evaluation and is easily comparable with existing rule-based methods.

## Related work

This section explores the current research into using machine learning algorithms to predict biodegradation reactions and pathways. We also summarise related research, such as papers tackling the generic chemical production prediction problem, as these methods can likely be applied to biodegradation prediction.

### Biodegradation prediction

Current methods for degradation prediction use a hybrid approach combining rule-based and machine-learning techniques [4]. Fenner et al. [5] found that the expert-crafted rules are often overly general. As such, when they are applied to novel chemicals, a combinatorial explosion of potential products occurs. Thus, previous research has focused on developing a method for predicting the likelihood of a reaction-rule combination occurring. This allows for pruning of the number of predicted products by removing those given a low probability [4]. Building on this foundation, we introduced enviPath, a comprehensive resource offering a biotransformation reaction database, pathway prediction system, and visualisation tools. This website made biodegradation reaction prediction significantly more available to researchers in the area [8].

Biodegradation reactions occur as part of biodegradation pathways. These pathways are represented by a graph where the root node chemical degrades into other chemicals, which themselves degrade. Existing studies report that data is often missing in biodegradation pathway datasets. This occurs as some reagents or intermediate products have half lives that are too short to be detected with current techniques and experimental procedures [20]. These complexities make evaluating pathway prediction methods challenging. These limitations were addressed by proposing a novel evaluation framework focusing on a method’s performance over a whole pathway instead of individual reactions and acknowledging the uncertainty in the experiment data [20]. To account for missing intermediate products, the evaluation metric does not punish a method for not immediately predicting a product as long as the actual product in the dataset is eventually given further down the predicted pathway. Additionally, the reactions in lower levels of the pathway contribute less to the overall score a method receives than those higher up to reflect the overall confidence in the data contained in the true pathway being correct. This holistic evaluation provides a more realistic assessment of biodegradation pathway prediction methods.

While the works above addressed biodegradation prediction assuming the existence of the expert rules, recent studies focused on automating the rule extraction required for the existing methods [6]. The enviRule system tackles the limitations of manual rule creation by automatically clustering and analysing biotransformation reactions, extracting and generalising rules in a data-driven fashion. This helps eliminate potential bias from manual rule curation and ensures a more comprehensive coverage of reactions. This automated approach complements the existing prediction methods by providing a source of higher-quality biotransformation rules compared to the existing expert rules. These rules further enhance the overall performance of biodegradation pathway prediction and is the current state-of-the-art method that we will be comparing our method against.

Wishart et al. [21] propose a hybrid rule and machine-learning method for predicting human metabolism pathways. In addition to rules, they also incorporate quantitative attributes such as mass and LogP. They find that combining the rules and other attributes produces a highly capable model for performing reaction and pathway prediction in the context of metabolism.

### Deep learning for product prediction

The past few years have also witnessed a surge in the application of deep learning methods for reaction prediction outside of biodegradation. This is thanks to the emergence of the large datasets that are required to facilitate the training of deep learning methods. The United States Patent & Trademark Office (USPTO) dataset, released in 2012, contains nearly one million unique chemical reactions data mined from the patents filed with the USPTO since 1976 [22]. This dataset has since been refined, and three main versions exist that are common benchmarks for prediction methods. These include the original unfiltered USPTO dataset, the USPTO_MIT dataset, which contains $\sim$400k reactions by removing all reactions containing stereochemistry and lastly, the USPTO 50k, which is a randomly selected subset often used for retrosynthesis prediction [15].

Initially developed for natural language processing, transformer-based methods emerged as the dominant architecture, achieving high accuracy on the USPTO dataset [22]. Schwaller et al. [14] demonstrated the effectiveness of transformers in predicting organic reaction outcomes. Expanding upon this work, Irwin et al. [15] proposed a self-supervised method of pre-training a transformer that could be refined to perform well on various chemical tasks. They train on 100 million chemicals, tasking the transformer with predicting an unmasked SMILES string from an augmented and masked version of the same SMILES string.

Transfer learning is a common practice in deep learning where a model is first trained on an extensive general dataset before being refined on smaller domain-specific datasets. This process has also been applied in product prediction with smaller datasets, achieving impressive performance. Wang et al. [17] and Zhang et al. [23] employed a transfer learning strategy with a transformer model to predict the small Heck and Baeyer-Villiger datasets. These studies highlight the excellent performance of deep learning models even in data-limited areas. Given the limited size of datasets present for biodegradation product prediction, our methods are inspired by these studies.

## Method

**Fig. 3:**
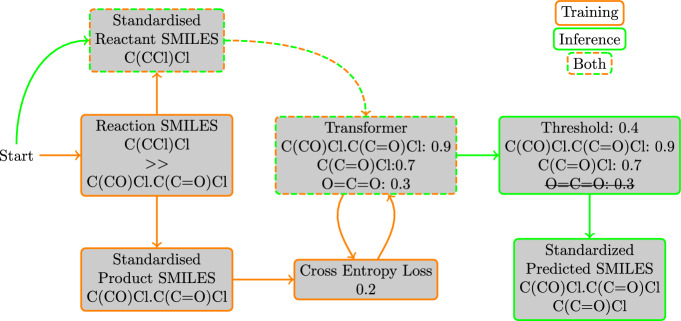
An overview of the enviFormer method. During training reactants and products are extracted from the train-set reactions and fed to the transformer. The transformers output is then compared with the true product to calculate the loss for the model to learn from. During inference a reactant SMILES is given to the transformer, which generates a list of potential products with associated probabilities. Depending on the threshold used different products will form the final set of output products

We formulate our problem as a sequence-to-sequence prediction task, aiming to predict a SMILES sequence, the biodegradation product(s), from a given input SMILES sequence, the reactant molecule(s). Therefore, enviFormer uses an encoder–decoder transformer model developed for natural language processing as it has been shown to perform well on sequence-to-sequence tasks [13]. An overview of enviFormer is shown in Fig. 3. This section details the pre-processing we apply to our data, our model’s architecture and our evaluation method.

### Pre-processing

We pre-process all molecule SMILES in our reactions using a series of standardisation rules. These rules do not change the molecule’s structure but alter the SMILES string to ensure that two molecules have equal SMILES. We explain each standardisation rule in Table 1, but most rules focus on standardising where hydrogen atoms are shown or hidden. On top of those rules, our pre-processing removes any stereochemistry information from the SMILES by removing @, / and $\backslash$ characters. We use these pre-processing steps to fairly compare with existing biodegradation methods that use such pre-processing [8]. We apply this standardisation to both the biodegradation data and the USPTO pre-training dataset to ensure the model is always presented with the same syntax.

**Fig. 4:**
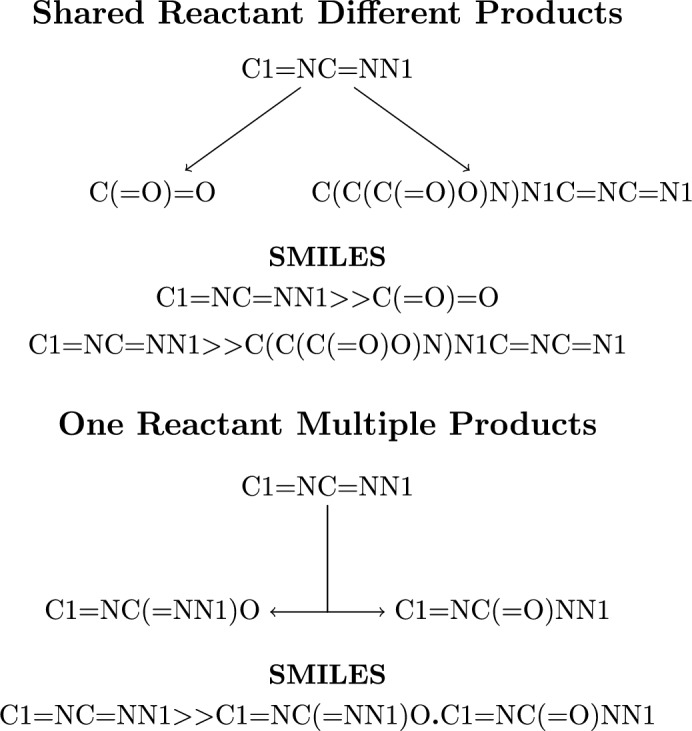
This example is different reactions 1,2,4-triazole undergoes taken from the Soil dataset. There are two cases of multiple products, separate reactions represented by two different SMILES and one reaction producing multiple products represented by one SMILES

In all the reaction datasets, there are instances where separate reactions share the reactant yet undergo different transformations, which produce different products. We do not merge these reactions; they are left separate reactions. In contrast, there are also individual reactions where multiple products are produced. This includes examples such as a molecule being split in two. For these instances, the products are separated by the dot character. Figure 4 gives an example of both these cases.

**Table 1:** The standardisation rules used to ensure a compound always has the same SMILES sequence

SMIRK description	SMIRK
Converting an enol group into a keto group.	[H][#8:2]-[#6:3]=[#6:1]>>[#6:1]-[#6:3]=[O:2]
Replacing Coenzyme A (CoA) group with a deprotonated hydroxyl group.	CC(C)(COP(O)(=O)OP(O)(=O)OCC1OC(C(O) C1OP(O)(O)=O)n1cnc2c(N)ncnc12)C(O)C(=O) NCCC(=O)NCCS[$(*):1]>>[O-][$(*):1]
Removing a hydrogen atom from a sulfur atom that is double-bonded to a phosphorus atom and either a sulfur or oxygen atom.	[H][S:1]-[#15:2]=[$([#16]),$([#8]):3]>>[S-:1]-[#15:2]=[$([#16]),$([#8]):3]
Removing a hydrogen atom from an oxygen atom bonded to a phosphorus atom.	[H][#8:1]-[#15:2]>>[#8-:1]-[#15:2]
Removing a hydrogen from a positively charged nitrogen atom that is bonded to a carbon atom.	[H][N+:1]([H])([H])[#6:2]>>[H][#7:1]([H])-[#6:2]
Deprotonating an oxygen atom connecting to a carbon atom which is triple-bonded to a nitrogen atom.	[H][#8:1][C:2]#[N:3]>>[#8-:1][C:2]#[N:3]
Removing a hydrogen from an oxygen atom that is in a carboxylic group.	[H][#8:1]-[#6:2]=[O:3]>>[#8-:1]-[#6:2]=[O:3]
Deprotonating an oxygen atom that is part of a nitro group.	[H][#8:1]-[#7+:2](-[*:3])=[O:4]>>[#8-:1]-[#7+:2](-[*:3])=[O:4]
Protonating an oxygen atom in an enolate structure.	[#6;A:1][#6:2](-[#8-:3])=[#6;A:4]>>[#6:1]-[#6:2](-[#8:3][H])=[#6;A:4]
Deprotonating an oxygen atom that is bonded to a phosphorus atom, except when the phosphorus is bonded to an oxygen atom with a negative charge.	[H][#8:1]-[$([#15]);!$(P([O-])):2]>>[#8-:1]-[#15:2]
Deprotonating an oxygen atom connecting to a meta-dinitrobenzene.	[H][#8:1]-[c:2]1[c:3][c:4][c:5]([c:6][c:7]1-[#7+:8](-[#8-:9])=[O:10])-[#7+:11](-[#8-:12])=[O:13]>>[#8-:1]-[c:2]1[c:3][c:4][c:5]([c:6][c:7]1-[#7+:8](-[#8-:9])=[O:10])-[#7+:11](-[#8-:12])=[O:13]
Removing hydrogen atoms from two hydroxyl groups in sulfuric acid.	[H][#8:1][S:2]([#8:3][H])(=[O:4])=[O:5]>>[#8-:1][S:2]([#8-:3])(=[O:4])=[O:5]
Deprotonating a hydroxyl group in a sulfonic acid structure bonded to a carbon atom.	[#6:1]-[#8:2][S:3]([#8:4][H])(=[O:5])=[O:6]>>[#6:1]-[#8:2][S:3]([#8-:4])(=[O:5])=[O:6]
Removing a hydrogen atom from an oxygen atom in a sulfoxide structure, forming a sulfonyl group.	[H][#8:3][S:2]([#6:1])(=[O:4])=[O:5]>>[#6:1][S:2]([#8-:3])(=[O:4])=[O:5]
Deprotonating an oxygen atom in a sulfinyl group structure.	[H][#8:1][S:2]([*:3])=[O:4]>>[#8-:1][S:2]([*:3])=[O:4]
Removing a hydrogen atom from a positively charged nitrogen atom.	[N+:1]([H])>>[N:1]
Removing a hydrogen atom from an oxygen atom bonded to an aromatic carbon atom.	[H][#8:1]-[c:2]>>[#8-:1]-[c:2]

### Transformer model

We use an encoder–decoder transformer model as it is designed for sequence-to-sequence tasks and has been shown to have superior performance to other sequence-to-sequence methods. The encoder–decoder transformer model works by learning the importance of each token in the input sequence relative to every other input token and output token. In doing so, it can predict appropriate output sequences given the input sequence [13]. To tokenise our SMILES sequences, we use a regular expression that breaks the sequence up. This tokenising largely splits the sequence into individual characters. However, atoms such as Chlorine will be made into one ‘Cl’ token. For example, Chloroacetaldehyde would tokenise from $\text {C(C}{=}\text {O)Cl}$ into $\text {C ( C }{= }\text {O ) Cl}$.

We use the same network architecture as previous work in the general product prediction area [14]. Our model hyperparameters are shown in Table 2. For adjusting the learning rate, we use the function suggested by the original transformer paper [13], given in Eq. 1 where $d=256$ and $warmup\_steps=4000$.

1 $$\begin{aligned} lr=d^{-0.5}\cdot \min (step\_num^{-0.5}, step\_num\cdot warmup\_steps^{-1.5}) \end{aligned}$$

**Table 2:** The parameters of our transformer model

Parameter	Value
Encoder layers	4
Decoder layers	4
Heads	8
Embedding dimension	256
Feedforward dimension	2048
Dropout	0.1
Embedding dropout	0.1
Max sequence length	276

We employ transfer learning akin to that used by Wang et al. [17] for Heck reaction prediction. Transfer learning involves first pre-training a model on a large general dataset and then using the learned weights as an initialisation point for further refinement on a target dataset. In our context, we use pre-training to teach the model common substructure changes that occur in chemical reactions and how to output syntactically valid output SMILES sequences. We use the full USPTO dataset containing $\sim 1{,}000{,}000$ reactions to perform pre-training. “Datasets” section gives more information about the datasets we use. Once pre-trained, the model is then refined on the smaller biodegradation datasets. Following similar research in the area, we do not freeze any of the model weights during transfer learning. We also ensure the learning rate is calculated using the final pre-training epoch in the learning rate function instead of resetting it as if we were training from scratch.

**Fig. 5:**
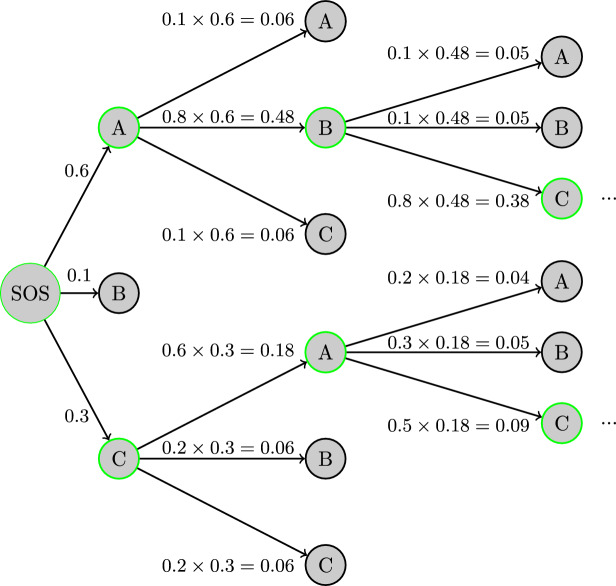
A beam decoding example using a simple ABC set of tokens to show how beam decoding is performed, starting with the SOS token. In this example, the beam width is two, and the two output sequences are ABC and CAC, with probabilities of 0.38 and 0.09, respectively

Our transformer model is an autoregressive model where each token is generated one at a time, starting from a special Start-of-Sequence (SOS) token, with respect to all the tokens generated thus far. We use beam decoding to create and rank the most likely candidate sequences, the same as the Molecular Transformer method [14]. In this decoding method, at each step, we assess a fixed number of potential subsequent tokens called the *beam width*, joining them with the sequences generated thus far and selecting the beam width of most likely new sequences to propagate to the next step. The probability of a sequence is calculated by multiplying the probability the model gave for each token in the sequence. At the end of the generation, we are left with the beam width number of sequences, each representing separate predicted product(s), each with a probability. Figure 5 gives an example of beam decoding. Each product sequence can be viewed as the model predicting alternative products that may be produced in different circumstances. One predicted sequence may contain multiple products, each separated by a dot. In this case, the model predicts that multiple separate products are produced during this one reaction.

We use a beam width of eight for our Single Generation evaluation discussed in “Single Generation evaluation” section and five for Multi Generation discussed in “Multi Generation evaluation” section. We do not use higher values for Single Generation as it becomes computationally expensive. We used a lower number for Multi Generation evaluation as it required more predictions and was too computationally intensive with higher beam widths. These sequences are associated with the probabilities given to them by the model. The threshold used to decide which sequences should be kept or rejected depends on whether high precision or recall is required. Lower thresholds will give higher recall, whereas higher thresholds will give higher precision. We test a range of thresholds during evaluation to generate Precision Recall (PR) curves.

### Evaluation methodology

**Fig. 6:**
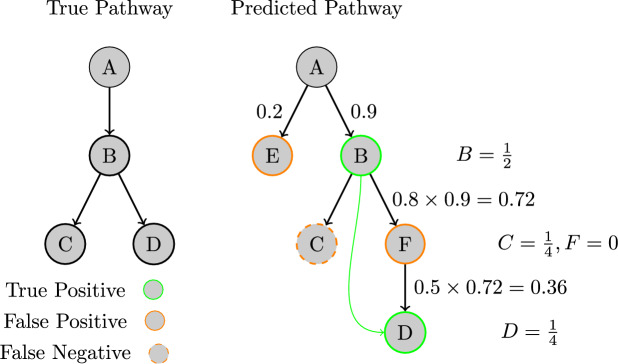
Multi Generation evaluation example. Note that compound F does not contribute to the score as it is an intermediate product and compound D gets its score adjusted as if it was an immediate product of compound B. With a threshold of 0.3, we get $Precision=(B+D)/(B+D)=1$, and $Recall=(B+D)/(B+C+D)=0.75$

Evaluating biodegradation product prediction is in itself a complex task. Applying transformer models to this problem leads to different equally valid evaluation metrics. As such, there are three ways we evaluate the method we propose to compare our models to existing methods best. These are Top-K, Single Generation and Multi Generation. They are summarised in Fig. 6 and Table 4. In all methods, we define a predicted product as equal to the actual products if the actual products are a subset of the predicted products. Treating products as sets allows predictions in different orders to the true products to still be considered equal. Using subset for comparison means predicting extra products does not penalise the model. We give an example of the implications of this in Table 3.

**Table 3:** Examples of how we define predicted products as being equal to actual products

Actual set	Predicted set	Equal
{CC=O, C(CO)N}	{C(CO)N, CC=O}	Yes
{C=O, CNC}	{CNC, C=O, O=C=O}	Yes
{C1COCCN1, C=O}	{C=O}	No

The first case is considered equal despite the order of the compounds. The second case is considered equal as all actual products are predicted and ignores the presence of a third compound. The third is not equal as the predicted set does not include all actual products

Additionally, for all tested methods, we ensure that the model outputs are standardised in the same way as the model inputs using the technique described in “Method” section. For all our evaluations, we use tenfold cross-validation splitting on biodegradation pathways. The training set is made by extracting all the reactions from the train-set pathways as we train on individual reactions, not pathways. We remove any reaction in the training set whose reactant appears as a reactant in the test set or whose product appears as a product in the test set as it is possible for different pathways to contain the same reactions.

**Table 4:** Single Generation example comparing Top-K and Single Generation evaluation

Correct reactions	Predicted reactions
$A\rightarrow B$	$A\rightarrow B:0.9 \space \space \space B\rightarrow C:0.9$
$B\rightarrow C$	$A\rightarrow E:0.2 \space \space \space B\rightarrow A:0.8$
$B\rightarrow D$	$A\rightarrow C:0.1 \space \space \space B\rightarrow F:0.7$
	$$A\rightarrow F:0.1 \space \space \space B\rightarrow D:0.5$$

Top-K	Single Generation
$K=3$	Threshold = 0.3
Precision = 2/3	Precision = 3/5
Recall = 2/3	Recall= 3/3

The Top-K and Single Generation evaluations focus on the model performance on individual reaction prediction. Subsequently, the test sets are extracted from the pathways in the same way as the training sets. We favour this over splitting by reactions as the compounds in one pathway are very similar, and by splitting on pathways, the compounds in the test set will be less similar to those in the training set. This allows us to see how our method performs on data further from the training distribution.

#### Top-K evaluation

First, to align with the other transformer-based methods, we use Top-K accuracy [14, 18, 24]. This is where we sort the transformer’s output sequences by their likelihoods and then take the top “k”. The accuracy for a given “k” is if the actual product is considered equal, by definition in “Evaluation methodology” section, to any of the top “k” predictions. The existing Top-K metric is insufficient here as it uses a simple sorting of probabilities, ignoring the possibility that although a sequence is the “k” most likely, its actual probability could be considerably smaller. For example, the third most likely sequence may be significantly less likely than the first two and thus should not be considered. Top-K is also insufficient, as it is commonly referred to as Top-K *accuracy*. However, the metric does not consider the drop in precision caused by predicting multiple products and resembles recall much more than accuracy. As such, it does not summarise the method’s performance well.

#### Single Generation evaluation

We use PR curves and the Area Under the Curve (AUC) for the second evaluation method. These align with the evaluation metric used on the existing biodegradation prediction methods [6, 20]. PR curves are preferred over the common ROC curves due to the issue of missing products discussed in “Biodegradation prediction” section. This issue means we do not have a strong notion of true negatives in the data and, therefore, can’t calculate the false-positive rate needed for a ROC curve. These curves are calculated by examining the likelihood of the transformer’s outputs. Therefore, unlike the general product prediction methods, we apply a threshold to determine whether to accept a prediction. By varying this threshold, we can create our desired PR curves that give us better insight into how our model is performing. Using this method, the model outputs get modeled like a binary classification problem, with any predicted sequence falling below the threshold being counted as a negative prediction and any above a positive prediction. If all positive predictions are invalid SMILES, the model will fail for the given input reactant.

#### Multi Generation evaluation

We also implement Multi Generation evaluation [20]. Here, the model is tasked with predicting the entire biodegradation pathway from the root compound. This means it must recursively predict new products from its previous prediction so long as the conditional probability of a pathway node, calculated from the root, is above a given threshold. If the model generates an invalid SMILES during the recursive prediction, the SMILES will be removed and will not be part of the next set of inputs. We can detect invalid SMILES as our SMILES standardisation will fail to give an output. This evaluation provides a score for the whole pathway instead of individual reactions. A key characteristic of this evaluation is that the weighting towards this score varies depending on the depth of the reaction in the pathway tree. Correctly predicting the first layer contributes more to the overall score than those further down the pathway. As shown in Fig. 6 this weighting equals $1/2^d$ where *d* is the depth in the pathway. The same goes for errors. However, we do not change the threshold in this way. Instead we rely on the conditional probabilities at higher depths being lower and thus more likely to fall below the threshold. It is worth noting that predicting intermediate products in the pathway does not impact the score as long as all true products are present and located on the correct pathway branch. Compound F in Fig. 6 is an intermediate compound and, as such, does not contribute negatively or positively to the method’s performance. This is a more realistic way to evaluate these models and better reflects the confidence in the data contained in the datasets [20]. Again, we use the same cross-validation split as the single-gen evaluation.

#### Datasets

We outline all the datasets used for training and evaluating our method in Table 5. These include:

- **USPTO** [22]. This is our pretraining dataset containing $\sim 1{,}000{,}000$ reactions. It was data-mined in 2012 from the patents containing chemical reactions that were filed with the United States Patent and Trademark Office since 1976. Subsequently, these reactions come from a wide variety of chemical contexts. Information about where each reaction originates is not readily available.
- **Soil** [11]. This dataset contains reactions extracted from the degradation of pesticides in different soil conditions submitted in draft assessment reports to the European Food Safety Authority.
- **Biocatalysis/Biodegradation Database (BBD)** [10]. This dataset contains enzyme-catalysed reactions and pathways of xenobiotic chemical compounds collected by experts.
- **Sludge** [12]. This dataset contains reactions representing the biodegradation of micropollutants in activated Sludge. It was created by collating reactions and pathways from the scientific literature.

**Table 5:** The statistics of our different datasets including number of reactions, pathways and unique compounds

Dataset	No. reactions	No. pathways	No. compounds	No. rules
USPTO	1,002,553	–	641,936	–
Soil	2446 (1798)	317	2609	80
BBD	1480 (1449)	219	1399	290
Sludge	500	184	1070	0

The number in brackets excludes reactions with carbon dioxide as the sole product, USPTO and Sludge do not have this occur. Note that the USPTO dataset does not contain pathways

## Experimental design

We split our dataset into train, validation, and test sets to train our transformer model. Our training set is used to update model weights, and the validation set controls early stopping to help prevent over-fitting. Finally, the test set is used to formally evaluate the model performance, giving the best performance estimation on unseen data. Before training on enviPath, we pre-train our transformer model using the full USPTO dataset. We split the dataset into three sets of 900k/50k/50k and used a batch size of 128. The model was trained for a maximum of 250 epochs; however, early stopping occurred at epoch 139 due to the validation loss failing to improve over the previous ten epochs. For refining the model, we reduced the batch size to 64 to increase model updates per epoch due to the small size of the biodegradation datasets. Additionally, we reload the learning rate from the last epoch of the pre-training process.

We are primarily interested in our model’s performance on biodegradation and in comparing it to the existing biodegradation models. Therefore, we give our model’s performance on each of the datasets present in enviPath, comparing our model to enviRule [6], and to the previous knowledge-data hybrid models [25]. Both enviRule and the knowledge-data hybrid models center on the use of an Ensemble of Classifier Chains (ECC) [26] to predict the probability of the transformation described by a rule actually occurring on a compound. EnviRule replaces the expert rules in the ECC with its own generated rules. We found the occasional instance where specific reactions would cause the existing methods to fail. In these instances, we count these reactions as incorrectly predicted. In the original enviRule paper, the authors extracted rules from all the reactions in the BBD and Soil datasets as they compared themselves to the expert rules created in the same manner. We have re-run the enviRule algorithm, extracting rules only from the training set to compare it to our method. Therefore, the results we report for enviRule differ from those reported in the original paper. For enviRule’s rule extraction algorithm, we used a radius of one and set the parameter *generalizeIgnoreHydrogen* to False, as this gave us the best results.

Before using the enviPath datasets, we remove all reactions with carbon dioxide as the sole product of this reaction. This is due to these reactions representing a complete breakdown of the reactant, which is often over idealised and is consistent with existing methods [6]. Additionally, as mentioned in “Pre-processing” section, all stereochemistry symbols are removed from the reactions.

Our enviPath dataset results are the mean from our tenfold cross-validation, and we use a single train test split for the USPTO dataset as it is not the focus of our evaluation and due to the computational limitations associated with running cross-validation on such a large dataset.

For comparing computational efficiency both methods were run on the same system with an AMD Ryzen 9 7950X 16-core CPU and a Nvidia RTX 4090. We evaluate our method both with CPU-only inference and GPU-accelerated inference. Our method is implemented in Python 3.11 using PyTorch 2.4 [27] for the transformer. For standardising SMILES, we use a Java implementation that is included with our Python code. We trained our model on one Nvidia RTX 4090 GPU, and with early stopping, this took approximately three days. All the enviPath data is available on the enviPath website, https://envipath.org/. Our code and the USPTO dataset are available online [28, 29].

## Results and discussion

This section provides our model’s performance on our three target datasets, Soil, BBD and Sludge. First, we look at coverage, a common metric used by existing biodegradation prediction methods. Secondly, we give the Top-K evaluation for all datasets, including our pretraining dataset, for comparison to existing transformer methods. We then show the benefits of using transfer learning compared to applying the pre-trained model. After, we look at the results from each of the enviPath datasets and compare our method to the existing methods. In this section’s figures and text, we refer to enviRule as the ECC model trained on rules generated by the enviRule algorithm and enviPath as the ECC model trained on existing expert rules in the enviPath database. In the enviPath dataset results, the Multi Generation results have significantly lower numeric values compared to the Single Generation evaluation. However, this does not mean a model performs poorly and should be interpreted as having different values produced by a different metric. Additionally, in the Multi Generation, the predictions had a maximum depth of seven if the method did not stop itself. Lastly, we give the computational efficiency results in “Runtime performance” section.

### Coverage

One advantage of our method compared to previous methods is its coverage. Coverage is the proportion of reactions in the dataset to which the model can be applied. Coverage does not look at whether a prediction is correct, simply that a prediction can be made. Both enviRule and the knowledge-hybrid method rely on transformation rules that cannot be applied to all reactions due to the syntactical limitations of rules. We find that enviRule achieves 77% coverage on the BBD dataset, 88% coverage on the Soil dataset, and 91% coverage on Sludge. In contrast, we can perform prediction on all the data, achieving 100% coverage on the Soil, BBD and Sludge datasets (Table 6). Additionally, there is no limitation on our model’s ability to perform predictions on any valid SMILES string, whereas the other existing methods may not be able to.

**Table 6:** The coverage that enviFormer, enviRule rules and enviPath rule sets achieve on the different datasets

Prediction method	BBD	Soil	Sludge
enviFormer	100.0%	100.0%	100.0%
enviPath rules	95.0%	45.5%	–
enviRule rules	77.2%	88.52%	91.55%

For enviRule rules are extracted from the whole of the respective dataset. As there are no expert rules for Sludge we do not calculate a coverage value

### Top-K results

Table 7 shows our Top-K evaluation using the metric traditionally used for evaluating transformer product prediction models. We include the performance of our pre-trained model on the USPTO dataset and find that our pre-trained model performs in line with the results reported by other papers [14]. We stop at Top-8 as that is the beam width we chose to use due to computational limitations as discussed in “Transformer model” section. For the Soil, BBD and Sludge results, we report the mean of the test sets from our cross-validation, where transfer learning was used to refine the pre-trained model. We note a substantial difference in performance as the Top-K increases on the enviPath datasets, with the most gain achieved by going from Top-1 at $\sim$20% to Top-2 at $\sim$26–32%, depending on the dataset. This occurs as many reactants in the enviPath datasets have multiple valid product sets, yet the model can only predict one set in Top-1, therefore automatically failing on the other product sets. This illustrates the short comings of the Top-K evaluation. The greater K values allow the model to predict multiple product sets and thus achieve a higher score. At the highest Top-8, we achieve an accuracy of around 50% on the enviPath datasets, over double that achieved at Top-1.

**Table 7:** Top-K evaluation of the different enviPath datasets and USPTO

Dataset	Top-1	Top-2	Top-5	Top-8
USPTO	74.4%	81.2%	85.8%	87.0%
Soil	20.1% ± 3.1	31.9% ± 3.5	46.9% ± 3.9	53.1% ± 4.5
BBD	20.4% ± 4.3	28.8% ± 5.0	41.0% ± 6.3	46.5% ± 6.4
Sludge	18.8% ± 6.4	26.7% ± 8.0	38.6% ± 8.4	45.5% ± 10.7

The Soil, BBD and Sludge are means of 10-fold cross-validation given with the standard deviation in percentage points

### Transfer learning

**Fig. 7:**
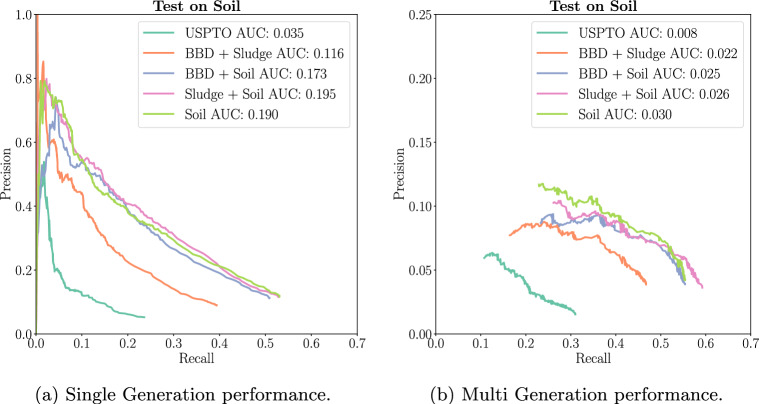
We show enviFormer’s performance on different training sets. Comparing the cyan USPTO line to other shows the benefits of transfer learning

Here, we show the benefits of using transfer learning for enviFormer’s performance. The cyan line in both plots of Fig. 7 shows enviFormer’s performance on Soil with USPTO as the sole training data. The other lines all included some part of the three enviPath datasets in the training data. These plots show that transfer learning benefits the model’s performance, with AUC values increasing up to 5.6x in Single Generation and 3.75x in Multi Generation. The orange line in both plots summarises the experiment where Soil is used only for testing with BBD and Sludge is used for transfer learning. Even in this case, the model benefits from training on biodegradation reactions that are not in the exact target domain, as it is still performing better than the cyan USPTO results (Fig. 7).

### BBD

**Fig. 8:**
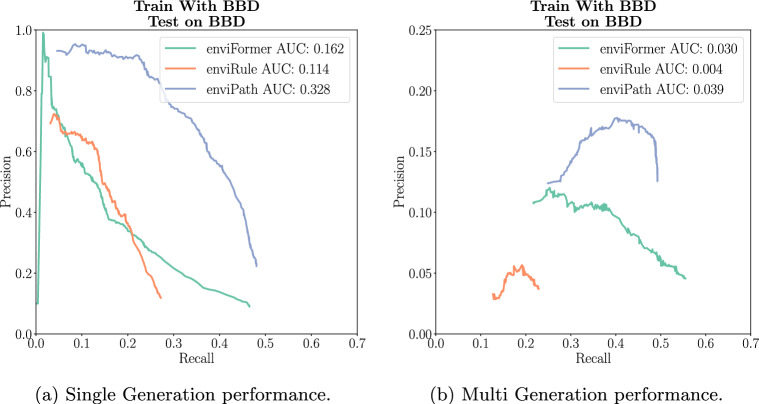
A comparison to the models trained with rules extracted by experts and extracted by enviRule on the BBD dataset

We find that the existing enviPath rules significantly outperform both our method and enviRule for the BBD dataset. In Single Generation evaluation, we achieved approximately the same recall as the existing rules of 0.48 but with lower precision. However, enviFormer outperforms enviRule, achieving a much higher recall and an AUC of 0.16 compared to enviRule’s 0.11. The Multi Generation results tell a similar story. The primary difference is that enviFormer achieves a slightly higher recall than the enviPath rules of 0.55 compared to 0.5, though still with lower precision. These results show that the expert rules have been highly tuned and perform very well on the dataset they were designed for (Fig. 8).

### Soil

**Fig. 9:**
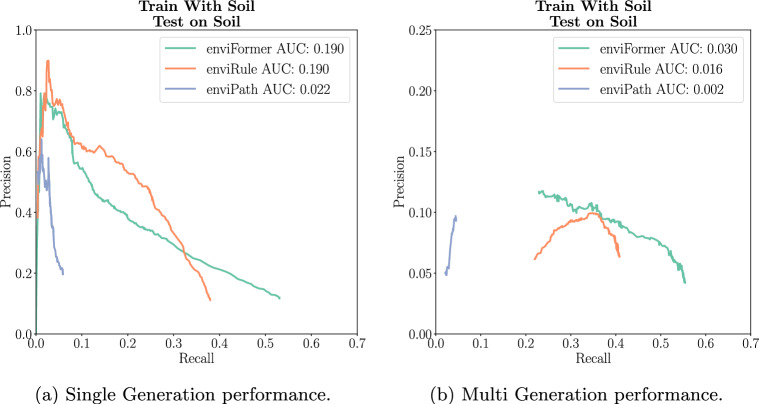
A comparison to the rules extracted by experts and extracted by enviRule on the soil dataset

Next, we evaluate our model on the Soil dataset. In Single Generation evaluation, enviFormer and enviRule have the same AUC, with enviRule achieving higher precision from a recall of 0.1 to 0.35 but a lower maximum recall of 0.4 compared to enviFormer’s 0.54. In Multi Generation, enviFormer outperforms enviRule in both regards, achieving an AUC of 0.03 compared to 0.016 with a higher maximum recall of 0.55 compared to enviRule’s 0.42. Both enviRule and enviFormer do significantly better than the Soil dataset expert rules currently available in enviPath. This suggests that in comparison to BBD the expert rules for Soil are significantly less refined and in general when rules are less refined enviFormer is the best method (Fig. 9).

### Sludge

Lastly, we evaluate our model on Sludge. Here, we highlight the performance of the different methods in the *leave one out* case. For the Sludge dataset, leaving one out means we train on the Soil and BBD reactions, leaving Sludge as a test set. Therefore, enviFormer is trained on all the reactions from Soil and BBD and enviRule extracts rules from the same set. For enviPath, this means using the expert rules from both BBD and Soil as one rule set. We perform these experiments as there are no expert rules for the Sludge dataset as of publishing.

**Fig. 10:**
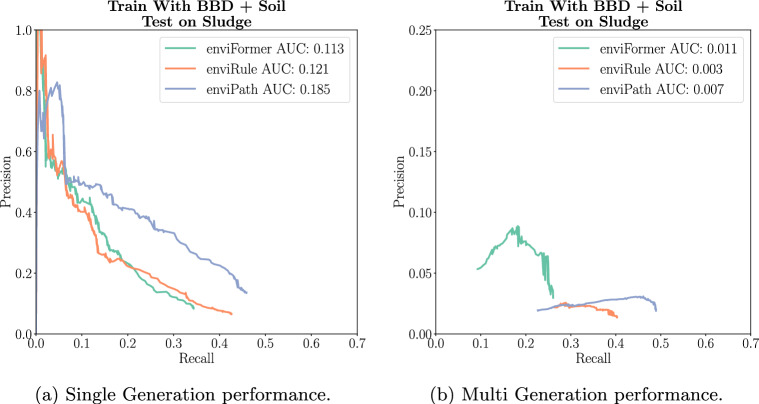
A comparison to the models trained with rules extracted by experts and extracted by enviRule from BBD and Soil and then evaluated on the Sludge dataset

In Single Generation, all three methods perform similarly. We see that enviPath comes out ahead with the highest recall of 0.46 and the highest AUC of 0.185. EnviFormer achieves precision similar to enviRule over most of the curve, however, its peak recall is 0.35 compared to enviRule’s 0.42. In the Multi Generation, enviFormer achieves the highest AUC of 0.011. This comes from a higher precision than the other methods despite the lower recall of 0.25. Like the Single Generation, enviPath achieves the best recall of 0.5. Overall, we see that transferring the existing BBD and Soil rules to Sludge can perform well in the Single Generation but leads to a very low precision in the Multi Generation scenario. Focusing on Multi Generation we see that enviFormer is the best method when looking at AUC and peak precision (Fig. 10).

### Runtime performance

We also evaluate the runtime of our method compared to enviPath’s existing hybrid rule machine learning based method. This evaluation uses all available reactions and pathways, excluding carbon dioxide reactions. Each batch size represents how many reactants are given to the model at a time and can highlight any performance gains from parallel processing. For pathway prediction, we limit the maximum width at each level to three and the maximum depth to four.

**Fig. 11:**
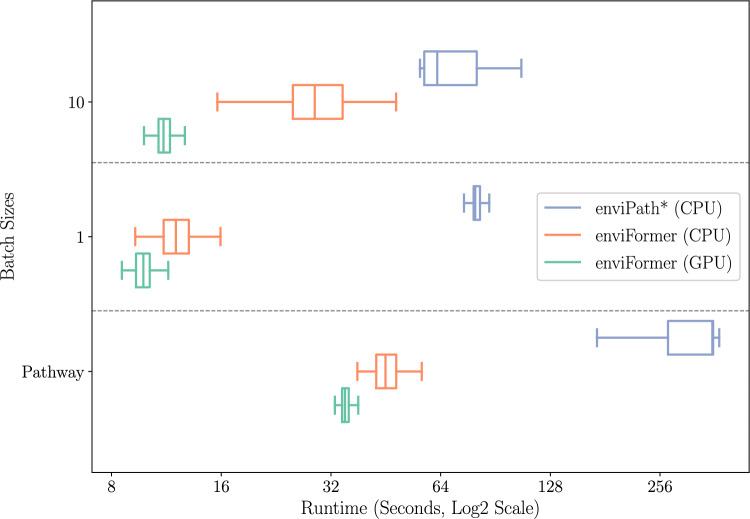
The runtime of enviFormer with (green) and without (orange) GPU acceleration compared to the hybrid rule based method (blue). Pathway represents the prediction time from the root. For the batch sizes the time reported is the time per batch. * We used a Python implementation of the Ensemble of Classifier Chains method utilised by enviPath [8] with 317 rules extracted by enviRule

Figure 11 shows that enviFormer is approximately eight times faster than the enviPath method in all the tested batch sizes. It also indicates that enviFormer performs best with GPU acceleration. However, if a GPU is unavailable, the CPU-only performance is only marginally behind. We include the pathway prediction time as predicting a whole pathway is a common task for prediction tools.

### Summary

Our study demonstrates that enviFormer provides significant improvements in coverage and computational efficiency. Achieving 100% coverage on the Soil, BBD, and Sludge datasets is a notable improvement over rule-based methods, indicating that enviFormer can be applied to a broader range of reactions and making it a more versatile tool for biodegradation prediction. Additionally, the computational improvements allow predictions to be performed significantly faster than existing methods. This is particularly beneficial when analysing batches of compounds or for predicting whole pathways.

We also conclude that transfer learning is crucial in enviFormer’s ability to predict biodegradation products. This suggests that the large general USPTO dataset does not contain reactions similar to those in biodegradation. Therefore, it is not sufficient to use off-the-shelf models.

Looking at the enviPath datasets, we see that in all three datasets, we either match or outperform enviRule, which we see as the primary competing method because it is also data-driven. We find that our method performs best on the Soil dataset. We hypothesise that this is due to a combination of the dataset being the largest of the three and having closely related reactions. The BBD results show that the expert rules perform significantly better than our method and enviRule. However, these rules have been developed over 20 years to suit this dataset specifically [30]. In comparison, the Soil rules have only been developed recently, and we can see they are not as refined from the results. Therefore, in scenarios where rules are unavailable or less refined, we summarise that enviFormer is the best-performing method. Our results indicate that those researching in this area should strongly consider a transformer-based method like enviFormer.

## Limitations and future work

Future research could look at data-augmentation techniques to help improve performance. Expanding the dataset with similar reaction datasets, such as the Braunschweig Enzyme Database [31] could also help improve our model’s performance. All current methods, including ours, do not incorporate environmental context into the predictions. While the availability of such information is limited, it could benefit prediction models. Similarly, information about the depth of the pathway or other pathway compounds is excluded.

Beyond transformers, other deep learning methods, such as graph neural networks, have seen success on the USPTO dataset. There is some evidence that these methods perform better on smaller datasets [32], and thus, applying them to the enviPath datasets could also be an avenue for future research.

## Conclusion

In conclusion, the prediction of biodegradation presents a complex and intricate challenge influenced by various environmental factors. Integrating machine learning techniques into chemical reaction prediction has significantly advanced our prediction capabilities. The need for predictive models is underscored by the growing awareness of the environmental consequences of chemical residues, prompting efforts to develop environmentally friendly chemicals. We show that transformer models can perform on par or better than the existing data-driven method enviRule while obtaining higher coverage. We also see that for datasets with less refined expert rules, like Soil, our model can outperform these expert rules. We also see a substantial computational efficiency gain compared to the existing rule-based methods. To our knowledge, our proposed method, enviFormer, is the first use of transformer models for biodegradation predictions.

## Data Availability

The data for reproducing the results is available at https://doi.org/10.5281/zenodo.13858534 and the code is available at https://doi.org/10.5281/zenodo.13858575.

## Abbreviations

BBD: Biocatalysis/biodegradation database
NLP: Natural language processing
SMILES: Simplified Molecular Input Line Entry System
USPTO: United States Patent & Trademark Office
SOS: Start-of-sequence
PR: Precision–recall
AUC: Area under the curve
ECC: Ensemble of classifier chains
